# Emerging School Readiness Profiles: Motor Skills Matter for Cognitive- and Non-cognitive First Grade School Outcomes

**DOI:** 10.3389/fpsyg.2021.759480

**Published:** 2021-11-23

**Authors:** Erica Kamphorst, Marja Cantell, Gerda Van Der Veer, Alexander Minnaert, Suzanne Houwen

**Affiliations:** Faculty of Behavioural and Social Sciences, Inclusive and Special Needs Education Unit, University of Groningen, Groningen, Netherlands

**Keywords:** school readiness, motor skills, whole child, person-centered approach, latent profile analysis, early childhood

## Abstract

A promising approach for studying school readiness involves a person-centered approach, aimed at exploring how functioning in diverse developmental domains conjointly affects children’s school outcomes. Currently, however, a systematic understanding lacks of how motor skills, in conjunction with other school readiness skills, affect a child’s school outcomes. Additionally, little is known about longitudinal associations of school readiness with non-academic (e.g., socioemotional) school outcomes. Therefore, we examined the school readiness skills of a sample of Dutch children (*N* = 91) with a mean age of 3 years and 4 months (46% girls). We used a multi-informant test battery to assess children’s school readiness in terms of executive functions (EFs), language and emergent literacy, motor skills, and socioemotional behavior. During the spring term of a child’s first grade year, we collected academic and non-academic (i.e., EFs, motor skills, socioemotional- and classroom behavior, and creative thinking) school outcomes. A latent profile analysis revealed four distinct profiles. Children in the “Parent Positive” (29%) profile were rated positively by their parents, and performed variably on motor and language/emergent literacy skills tests. The second profile–“Multiple Strengths” (13%)–consisted of children showing strengths in multiple domains, especially with respect to motor skills. Children from the third profile–“Average Performers” (50%)–did not show any distinct strengths or weaknesses, rather displayed school readiness skill levels close to, or just below the sample mean. Finally, the “Parental Concern” (8%) profile was characterized by high levels of parental concerns, while displaying slightly above average performance on specific motor and language skills. Motor skills clearly distinguished between profiles, next to parent-rated EFs and socioemotional behavior, and to a lesser extent emergent literacy skills. School readiness profiles were found to differ in mean scores on first grade academic achievement, parent- and teacher-rated EFs, motor skills, parent-rated socioemotional functioning, and pre-requisite learning skills. The pattern of mean differences was complex, suggesting that profiles could not be ranked from low to high in terms of school outcomes. Longitudinal studies are needed to disentangle the interaction between emerging school readiness of the child and the surrounding context.

## Introduction

Ample evidence supports the notion that a child’s skills at school entry (i.e., their school readiness) set the stage for a successful and adaptive school career (Snow, 2006; Duncan et al., 2007; Valiente et al., 2021). It is thus not surprising that early childhood school readiness screening has received increased attention from policy makers. According to the Incheon Declaration and Framework for Action, ensuring that each and every child is prepared to thrive in school is a goal that early childhood policy makers should view with the utmost urgency and importance (UNESCO, 2015). Meeting this goal calls for a thorough assessment and understanding of variation in early childhood school readiness (Hirsh-Pasek et al., 2005). Such insights can subsequently inform the design of tailored early childhood education and care programs, aimed at boosting each child’s prospect of a successful transition into the formal educational system.

An up-and-coming school readiness research strand that focusses on the individual child as a whole, is the person-centered (PC) approach. It explores how functioning in diverse developmental domains conjointly affects children’s school career and outcomes (Bergman and Magnusson, 1997; Marsh et al., 2009). Such a holistic perspective aligns with a recent perspective on whole child education, which seeks to support and nurture a child’s development in all areas. The ultimate goal being to realize each child’s full potential and develop them as future citizens of the 21st century (Slade and Griffith, 2013; OECD, 2018). Empirical evidence endorsing the PC approach for research on school readiness stems from a growing body of studies that have found differences in school success between subgroups of children with distinct school readiness profiles (e.g., McWayne et al., 2009; Denham et al., 2012; Halle et al., 2012; Mascareño et al., 2014). However, hardly any of these previous studies included the motor domain, although both gross and fine pre-school motor skills have been found to be pivotal for school success (Cameron et al., 2016). Therefore, the present study aimed to extend the current knowledge base by examining the role of the motor domain as part of school readiness profiles. Additionally, we expanded the whole child perspective to the conceptualization of school success. That is, while many previous studies applied a narrow focus on only “core” academic skills, we implemented a broad assessment comprising of cognitive (e.g., literacy) and non-cognitive (e.g., motor or socioemotional) skills that have been linked to, and deemed necessary for school success and functioning in later life and society (Pagani et al., 2010; Kim et al., 2016).

### School Readiness as Cornerstone for a Successful School Career

When defining school readiness, it is important to specify who or what is supposed to be “ready,” as the concept has been used interchangeably to indicate a child’s skills at the onset of formal schooling, a school’s readiness to support a child, as well as the readiness of family and environment to stimulate early development (Keating, 2007). The present study is aimed at the former, for which a commonly accepted description emphasizes the multidimensional nature of this concept (Snow, 2006; Blair and Raver, 2015; Bell, 2017). Indeed, within a variable-centered framework both cognitive and non-cognitive skills have been studied for their unique and/or relative contribution to school success (La Paro and Pianta, 2000; Denham, 2006; Duncan et al., 2007; Pagani et al., 2010). Findings have been mixed, on one hand, with some studies insinuating that cognitive aspects of school readiness (such as basic language and reasoning abilities) are most decisive for later learning (La Paro and Pianta, 2000; Duncan et al., 2007). On the other hand, studies have shown that a broader constellation of early child capacities, including non-cognitive abilities such as motor skills and social competence, is predictive of later school achievement (Denham, 2006; Pagani et al., 2010; Pagani and Messier, 2012). However divergent, most findings corroborate the notion of school readiness as constituting the cornerstone of a child’s positive school adaptation. Lasting effects have even been found well into the late school career (Stipek, 2001; Entwisle et al., 2005), which might be explained by so-called cascading or cumulative mechanisms.

### A Person-Centered Approach to School Readiness

The last decade of school readiness studies has been characterized by a shift from a variable- to a PC approach (e.g., McWayne et al., 2009; Denham et al., 2012; Halle et al., 2012; Mascareño et al., 2014; Iruka et al., 2018, 2020). Summarizing findings and drawing inferences across this emergent body of literature is challenging as studies are characterized by a wide variety in samples (e.g., a specific Head Start sample or a community sample), age (ranging from 3 to 7 years), and indicators of school readiness (e.g., a focus on socioemotional skills versus cognitive and pre-academic skills). Nevertheless, from these studies some common findings have emerged, which highlight important insights that can be gained when using a PC-approach.

First, most studies identified subgroups of children with unique patterns, or profiles, of school readiness skills. Importantly, profiles were often not (or not only) distinctive in terms of the level of school readiness skills included (e.g., one profile with above average functioning on all skills, and one profile of low functioning over the whole range of school readiness skills). That is, often so-called mixed profiles were delineated, that consisted of a combination of relative strengths and weaknesses (McWayne et al., 2004, 2012; Konold and Pianta, 2005; Hair et al., 2006; Smeekens et al., 2008; Bulotsky-Shearer et al., 2010; Denham et al., 2012; Halle et al., 2012; Quirk et al., 2013; Mascareño et al., 2014; Abenavoli et al., 2017; Justice et al., 2017; Collie et al., 2018; Iruka et al., 2018; Martarelli et al., 2018; Sandilos et al., 2019; Tavassolie et al., 2020). For example, Justice et al. (2017) examined kindergarten readiness profiles in a sample of low-income children from rural areas. Four profiles emerged, of which two were characterized by patterns of diverse skill levels. That is, an “academic risk” profile was found for a subgroup of children who scored below average on language, literacy, and math skills, while showing (slightly above) average levels of socioemotional skills and learning-related behaviors. Additionally, children classified as the “sociobehavioral risk” subgroup showed the converse profile of (slightly above) average pre-academic skills and below average sociobehavioral skills.

Second, many studies explored the predictive validity of school readiness profiles and found them to be differentially and meaningfully related to academic and/or other (mainly cognitive) school outcomes (McWayne et al., 2004, 2012; Konold and Pianta, 2005; Hair et al., 2006; Bulotsky-Shearer et al., 2010; Denham et al., 2012; Sabol and Pianta, 2012; Quirk et al., 2013; Mascareño et al., 2014; Abenavoli et al., 2017; Collie et al., 2018; Martarelli et al., 2018; Tavassolie et al., 2020). In general, high-functioning (i.e., high performance on all profile indicators) profiles were associated with high performance on school outcomes, whereas children in low-functioning profiles were likely to be characterized by less favorable school functioning (e.g., McWayne et al., 2004; Hair et al., 2006; Bulotsky-Shearer et al., 2010; Denham et al., 2012; Collie et al., 2018). More interesting in terms of the usefulness of a PC approach, are the complex ways in which mixed profiles were associated with concurrent or future school outcomes. For example, low(er) cognitive abilities are not necessarily associated with low(er) academic outcomes in all children. This is illustrated by the study of Martarelli et al. (2018) on kindergartner’s executive functions (EFs) and social skills profiles. In their study, children with low EFs in combination with moderate social skills performed in the average range on future academic outcomes.

### Importance of the Motor Domain as Part of School Readiness

Notwithstanding their contributions to establishing the relevance of a PC approach, previous PC-studies have left several key issues in the field of school readiness underexplored. First, as far as we know few PC-studies have incorporated motor skills into school readiness profiles, which is remarkable as variable-centered studies have found associations between pre-school motor skills and school success (e.g., Grissmer et al., 2010; Cameron et al., 2016; de Waal, 2019; McClelland and Cameron, 2019; Ricciardi et al., 2021). More specifically, gross motor skills have been found to be pivotal for social engagement (Bart et al., 2007; Escolano-Pérez et al., 2020), as well as academic skills such as math and reading (Donnelly et al., 2016; Ricciardi et al., 2021). Fine motor skills, such as visuo-motor and visuo-spatial integration skills, have been consistently associated with performance on literacy and math tests (Carlson et al., 2013; Chang and Gu, 2018; McClelland and Cameron, 2019; Suggate et al., 2019; Chandler et al., 2021; Mohamed and O’Brien, 2021). Several mechanisms have been proposed to explain the established links between pre-school motor skills and later school outcomes. First, the automaticity hypothesis posits that being more competent in a certain skill results in a more automatic execution of that skill, thereby freeing up attentional resources for other demanding tasks (Floyer-Lea and Matthews, 2004). Furthermore, the functionalism view entails that the better a child has developed its motor skills, the more it can benefit from environmental affordances, opening up a whole array of new learning opportunities (Mohamed and O’Brien, 2021). Both mechanisms suggest that well-developed motor skills equip a child with essential assets for learning. While these accounts inform us about how motor skills might be important for school outcomes, these mechanisms cannot be readily tested within a PC-approach. Instead, what can be explored is how motor skills, in combination with other school readiness skills, affect school outcomes. In this respect, so-called compensatory processes for the combination of cognitive and socioemotional skills have been found in previous PC-studies (Konold and Pianta, 2005; Hair et al., 2006). For example, Konold and Pianta (2005) found that strengths in terms of cognitive skills compensated for social deficits and externalizing behavior, when considering first grade academic outcomes. Speculatively, motor skills could also be part of such a (long-term) compensation mechanism. For example, fine motor skills and self-regulation have been found to co-develop, and being skilled in one might act as a buffer against deficiencies in the other (e.g., Oberer et al., 2017; McClelland and Cameron, 2019; Chandler et al., 2021).

Only a handful of PC studies did incorporate motor skills. Two of these included motor skills as part of one overlapping “physical health” indicator. Whilst thus not conclusive about the role of motor skills in particular, study results provided preliminary support for the possible role of motor skills concerning a child’s school readiness (Hair et al., 2006; Quirk et al., 2013). In a community sample of 5-year-olds, Hair et al. (2006) studied school readiness profiles based on four indicators, that is, physical health, socioemotional-, language-, and cognitive skills. Physical health was assessed by ratings of health status, healthy weight, and fine and gross motor skills, each receiving a classification of either “on track” or not. By means of a k-means cluster analysis, four distinct profiles were identified in terms of physical health level (range: above average to well below average). Moreover, two profiles emerged with similar levels of language and cognitive skills, yet one of these profiles was characterized by above average physical health level, while children in the other profile displayed significantly below level physical health. Notably, the former profile was associated with better first grade child outcomes as compared to the latter, suggesting that a better overall physical health status mitigates relative weaknesses in the cognitive domain on the long-term. In a study on US kindergarten Latino(a) children, three items regarding physical health (including a teacher rating of fine motor and general coordination skills) were included as part of comprehensive kindergarten readiness profiles (Quirk et al., 2013). Study findings align with those of Hair et al. (2006) in terms of the usefulness of a physical health indicator to separate between school readiness profiles.

Unlike Hair et al. (2006) and Quirk et al. (2013), the health domain, which included parent and teacher ratings of fine and gross motor skills next to general health- and special needs status, hardly differentiated between the readiness profiles of 4-year-old Head Start children (Halle et al., 2012). However, the authors suggested this might be due to the limited variation on this indicator in their study sample. Furthermore, Tavassolie et al. (2020) found that fine and gross motor skills consistently displayed similar performance levels as compared to skills from the cognitive domain, within each profile that they identified. Together, these motor and cognitive skills clearly distinguished between school readiness profiles of 4-year-old children from a low-income, ethnically diverse sample. Furthermore, profile membership was associated to third grade academic achievement and retention rate. Differences between profiles, and thus the main drivers of third grade performance, seemed to be mostly stemming from the type of test and informant used (e.g., direct assessment, versus behavior ratings by parents or teachers). As a result, based on their findings, it remains unresolved if and how motor skills in combination with other school readiness skills, might add to a successful school career. In sum, the few empirical studies that included skills from the motor domain do not yet provide a systematic understanding of how motor skills in conjunction with other school readiness skills, affect a child’s transition into the formal education system.

### A “Whole Child” Approach to School Success

Another aspect to consider when studying school readiness is how it predicts later school outcomes. Commonly, researchers have focused on the association between school readiness and core academic skills, such as reading and math (e.g., Konold and Pianta, 2005; Quirk et al., 2013; Martarelli et al., 2018). Yet, it is strongly debated what holds as “school success” and thus what should be considered as a criterion measure for determining favorable constellations of children’s ability to thrive in an educational setting (Bowles et al., 2001; Biesta, 2010). In other words, it is suggested that–next to core academic skills-, other cognitive- (EFs), and non-cognitive (socioemotional and classroom behavior, motor skills, and 21st century skills) skills are linked to, and necessary for long-term school success and functioning in later life and society (Pagani et al., 2010; Kim et al., 2016). Concerning 21st century skills, different frameworks and conceptualizations exist which seem to converge on several basic skills (Ananiadou and Claro, 2009; Battelle for Kids, 2009). These include problem solving, communication, collaboration, digital literacy, critical thinking and creativity, which are all deemed pivotal for successful learning and thriving in the 21st century society (Thijs et al., 2006). The current study focused on creativity, as this has already been linked to academic achievement and cognition, and valid measures are available for primary school aged children (e.g., Collard and Looney, 2014; Beghetto et al., 2016). Although several studies included some non-academic aspects of school success (Hair et al., 2006; Bulotsky-Shearer et al., 2010; Denham et al., 2012; McWayne et al., 2012; Sabol and Pianta, 2012; Mascareño et al., 2014), to our knowledge we will be the first to employ a whole child perspective to the conceptualization of school success. This approach also aligns with the Incheon Declaration and Framework for Action’s focus on both cognitive- and non-cognitive development (UNESCO, 2015).

### The Current Study

In the current study, we set out to extend the PC school readiness literature by acknowledging the importance of motor skills in combination with other school readiness skills, and a whole child perspective on the conceptualization of school success. More specifically, the following research questions were addressed: (1) Which school readiness profiles can be distinguished within 3-year-old children, when EFs, language and emergent literacy skills, motor skills, and socioemotional behavior are incorporated simultaneously? and (2) How do distinct patterns of school readiness skills predict cognitive (i.e., academic achievement and EFs), and non-cognitive (i.e., motor skills, socioemotional and classroom behavior, and creativity) outcomes in first grade? We aimed to address these research questions by assessing different school readiness skills at age 3, and a broad range of first grade school outcomes. As far as we know, we are one of the first to explore school readiness profiles at an age well before children enter any form of more or less formal early childhood education (e.g., pre-K or kindergarten curricula). Doing so holds the potential for optimally preparing each child before the transition to a structured and challenging school setting, rather than remedying an already existing gap after this crucial step in a child’s early life. Based on previous studies that did include skills from the motor domain, we expected to identify between four and six school readiness profiles (Hair et al., 2006; Halle et al., 2012; Quirk et al., 2013; Tavassolie et al., 2020). An additional expectation was to identify a profile characterized by an overall pattern of unfavorable school readiness performance, putting children in this profile at risk of a less successful school career. Given limited and mixed findings concerning the role of the motor domain and differential associations to both cognitive and non-cognitive school outcomes, we refrained from formulating specific hypothesis on these matters.

## Materials and Methods

### Participants

The current study’s sample was part of a larger longitudinal research project: “MELLE” (Motor skills, Executive functions, Language, and LEarning outcomes in preschool children; see also Houwen et al., 2019), in which 3- to 5-year old children were followed regarding their EFs, language/emergent literacy skills, motor skills, and socioemotional behavior, and first grade school outcomes (Houwen et al., 2019). Children were recruited from kindergartens and day-care centers, and via social media, flyers and posters distributed at supermarkets, stores, and playgrounds. Inclusion criteria were: (a) no signs of a medical condition (e.g., heart disease), neurological disorder (e.g., cerebral palsy), intellectual disability, or physical disability (e.g., club foot), (b) normal hearing and normal or corrected to normal vision, (c) being able to follow the test instructions, and (d) having parents/caretakers who have sufficient proficiency in written Dutch to be able to complete the questionnaires.

Given our focus on school readiness before the start of any formal schooling, we selected a subsample of children who were 3-year-old at their first measurement occasion. Data on the school readiness of these children were collected between April 2016 and May 2019. The final sample consisted of 90 children (54.4% boys), aged 35–47 months old (*M* = 40.9 months, *SD* = 3.5 months). Socioeconomic status (SES), based on maternal educational level (Ursache and Noble, 2016), was unequally distributed across low SES (3.4%), intermediate SES (17.2%), and high SES (79.3%). Participants resided in the northern part of the Netherlands, and came from a variety of community types, ranging from rural to urban areas.

School outcome data were collected for a subsample of 47 children, whose parents gave renewed consent for participation in the extension of the original study, concerning first grade data-collection of the MELLE-project. Known reasons for attrition (48%) were: COVID-19 concerns, children unwilling to further participate; assessment not fitting the family schedule; children skipping a grade and already being in second grade; inability to reach some parents from the original sample by phone or mail. Differential attrition analysis revealed that children that did participate in the first grade school outcome data-collection did not differ from the drop-out group in terms of gender (χ^2^ (1) = 0.45, *p* = 0.150 *V* = 0.07), age (*t* (88) = −1.86, *p* = 0.07, *r* = 0.19), or most likely profile membership ((χ^2^ (3) = 0.52, *p* = 0.91 *V* = 0.08). Attrition was associated to SES (χ^2^ (2) = 11.41, *p* = 0.002 [Fisher’s Exact test], *V* = 0.36). The drop-out group consisted of a higher percentage of children from a medium SES (32%) as compared to the children that did participate in first grade (4.3%). During the first-grade data-collection children were 6–7 years old (*M* = 81.5 months, *SD* = 3.75; 51% boys).

### Measures

For readability, we only provide a short description of tests and questionnaires that were administered according to a standardized manual and test protocol. For further details concerning administration and psychometric properties we refer to the corresponding manuals and additional sources we cited.

#### Three-Year-Old School Readiness Indicators

##### Performance-Based Measures of Executive Functions

Three performance-based EFs measures were used for inhibition and two for working memory. That is, verbal inhibition was measured by the Day/Night task (Gerstadt et al., 1994), and fine and gross motor inhibition were measured by the Hand Tapping (Diamond and Taylor, 1996) and Head-to-Toes (Cameron Ponitz et al., 2008) task, respectively. Specifics of each task’s administration can be found in Houwen et al. (2019). Concerning the Head-to-Toes task, we only used the first series of 10 test trials. The Forward Corsi Block task (Pickering et al., 1998) was administered as a measure of visuo-spatial working memory, while the Forward Digit Recall task (Gathercole and Pickering, 2000) was used to assess verbal working memory. The EF tasks were age adequate and have shown acceptable to good test score reliabilities (Alloway et al., 2009; Rhoades et al., 2009; Allan and Lonigan, 2011; Müller et al., 2012; Meador et al., 2013). Based on previous studies on the structure of EFs with 3-year-olds (Wiebe et al., 2008; Willoughby et al., 2012; Houwen et al., 2019), we expected the performance-based EFs to be best represented by a one-factor model. Therefore, we carried out a confirmatory factor analysis (CFA) and found a good fit for the one-factor model (see online Supplementary Materials, Section 1). The estimated EFs factor score was used in subsequent analysis, whereby a higher score indicated better EFs.

##### Rating Scale of Executive Functions

Parents or caregivers completed the Dutch translation of the Behavior Rating Inventory of Executive Function–Pre-school version (BRIEF–P) (Gioia et al., 2005), a 63-item standardized rating scale that assesses different aspects of EF in children 2;0–5;11 years. Corresponding to the performance-based EFs subdomain measures, only the subscales Inhibition and Working Memory were used in the present study. Age- and sex-specific T-scores (*M* = 50, *SD* = 10) were used in subsequent analyses, in which higher scores indicate greater executive dysfunction. The Dutch version of the BRIEF-P has shown good reliability and validity (van der Heijden et al., 2013).

##### Language and Emergent Literacy Skills

Three performance-based tests were used to measure language and emergent literacy skills. First, the Pseudowords Repetition test of the Schlichting Test for Language Production (Schlichting and Lutje Spelberg, 2010) measures phonological processing skills. The test has shown excellent internal consistency and inter-rater reliability, but low test–retest reliability (Schlichting and Lutje Spelberg, 2010). Furthermore, construct validity is supported by the fact that raw test scores were associated with age and other language subtests from the Schlichting Test for Language Production. Raw scores consisted of number of accurate responses on both existing and non-existing words (0–40). Second, the Rapid Naming Pictures subtest of the Test for Continuous Naming and Word Reading (van den Bos and lutje Spelberg, 2010) was used to measure naming speed. The total raw score was the overall time it took to complete the task in seconds. Sufficient reliability and validity have been established in a sample of 8–14 year-olds (van den Bos and lutje Spelberg, 2010). Finally, the Receptive Vocabulary subtest of the Dutch version of the Wechsler Pre-school and Primary Scale of Intelligence Third Edition (WPPSI-III-NL) (Wechsler, 2009, 2010) was used to measure receptive vocabulary. The total score is the sum score of correct responses, which were converted into age-standardized scores (*M* = 10, *SD* = 3). Sufficient-to-good reliability for the WPPSI-III-NL and evidence for good content, construct, and criterion validity of the instrument have been reported (Wechsler, 2009, 2010).

##### Motor Skills

Motor skills were assessed with age band 1 from the Dutch version of the Movement Assessment Battery for Children Second Edition (MABC-2) (Henderson et al., 2010). This test consists of three motor components: Manual Dexterity (three items), Aiming and Catching (two items), and Balance (three items). The raw scores of each item were recoded into an item standard score, which uses correction for age. Subsequently, these item standard scores were summed into a total standard score (range 1–19, *M* = 10, *SD* = 3) per component (i.e., Manual Dexterity, Aiming and Catching, Balance). These component scores were used as separate school readiness indicators in further analyses. The psychometric properties of the MABC-2 suggest that it is a valid and reliable measure to be used in 3-year-old children (Ellinoudis et al., 2011; Smits-Engelsman et al., 2011).

##### Socioemotional Behavior

Parents completed the Dutch translation of the Strengths and Difficulties Questionnaire (SDQ) (van Widenfelt et al., 2003), which assesses five domains of socioemotional functioning, including four scales indexing socioemotional behavior problems (Emotional Problems, Conduct Problems, Hyperactivity, and Peer Problems) and one scale indexing socioemotional strengths (Prosocial Behavior). Subscale scores were calculated by summing the corresponding five item scores (range 0–10). As recommend by Goodman et al. (2010), we used the Externalizing- (sum of Conduct Problems and Hyperactivity) and Internalizing (sum of Emotional Problems and Peer Problems) subscales, where higher scores indicate more socioemotional behavior problems. Additionally, we included the subscale Prosocial Behavior, whereby higher scores reflect a higher level of prosocial skills. In general, sufficient reliability, and construct and concurrent validity have been shown in studies assessing Dutch samples (Theunissen et al., 2013, 2015; Maurice-Stam et al., 2018). It should be noted though that a poor internal consistency has been found for the Internalizing subscale (α of 0.47) in one study (Maurice-Stam et al., 2018).

#### First Grade School Outcomes

We applied a multi-method, and multi-informant approach to the assessment of school outcomes. That is, we administered a combination of performance-based tests and proxy questionnaires from both parents and teachers. A major part of the performance-based tests consisted of subtests of the Dutch version of the Intelligence- and Developmental Scales Second Version (IDS-2) (Grob and Hagmann-Von Arx, 2018). For most of these subtests, raw scores were converted to *z*-scores, based on monthly age-norms, and subsequently to a standardized score (*M* = 10; *SD* = 3). Modifications of this procedure, if any, will be described per subtest.

##### Academic Achievement

Performance on academic subjects (i.e., math, language, and literacy) was assessed by a combination of individually administered tests and nationally standardized instruments. For the latter we collected results of nationwide tests for monitoring yearly progress of Dutch primary school students (National Institute for Educational Measurement [in Dutch: CITO], 2015). These tests were administered to the whole-class in January of each child’s first grade academic year. Raw test scores are first converted into normed, continuous ability scores. These scores can be classified into five percentile groups relative to the general Dutch population, which we used for further analyses.

**FIGURE 1:**
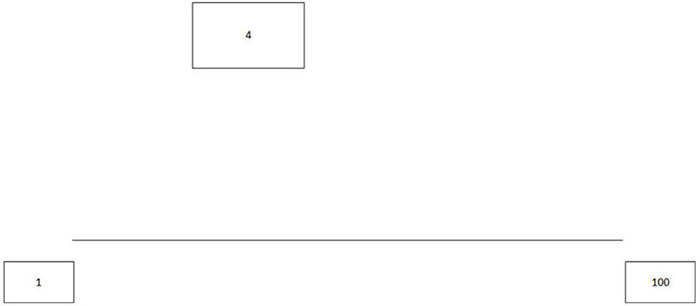
Example item of the number line task.

*Math*. The IDS-2 subtest Logical-Mathematical Thinking was aimed at measuring number sense and basic mathematical skills. This test has been found to display good reliability, as well as convergent and discriminant validity (Grob and Hagmann-Von Arx, 2018). Furthermore, we administered a bounded version of the number line task (NLT) (Siegler and Opfer, 2003), to further tap into number sense. The NLT was 19.8 cm long, and ranged from 1 to 100. The target number was presented in the center above the number line (see Figure 1) and also read aloud to the child. The child was asked to draw a vertical dash on the number line to mark their estimated position of the target number. Target numbers were derived from the study of Friso-van den Bos et al. (2015), and presented in one of five randomized orders. Accuracy of number line estimation was established by computing the percentage absolute error (PAE = absolute error/length of line ^∗^100) per target number (e.g., van der Weijden et al., 2018). Subsequently, individual target number PAE’s were averaged to produce a mean PAE per participant, where a lower mean PAE indicated more accurate number line estimation. Reliability of the NLT has been found to be sufficient (Link et al., 2014) and convergent validity has been established in several studies (Sasanguie et al., 2012a, b). In addition to these individually administered tests, we collected scores on the national CITO mathematics tests (Janssen et al., 2015). This test entails mostly word problems which call upon several skills, that are part of the Dutch first grade curriculum, such as basic operations and time. The CITO mathematics test has been found to be reliable and display sufficient construct validity (Janssen et al., 2015).

*Language and Literacy*. Oral language skills were assessed by the IDS-2 subtests Receptive Language and Expressive Language. We used the standard scores per subtest for further analyses. Reliability and convergent validity of these subtests have been established in a Dutch sample (Grob and Hagmann-Von Arx, 2018). Two CITO subtests were administered to assess reading and spelling skills. First, the 3 min test (in Dutch: Drie-Minuten-Toets; DMT) is aimed at examining technical reading (van Til et al., 2018). Second, the Spelling test examines active spelling of non-verbs (Tomesen et al., 2017). Reliability and convergent validity of both tests has been evaluated as sufficient (Tomesen et al., 2017; van Til et al., 2018).

*Data Summarizing*. Given many statistically significant correlations between academic achievement variables (see online Supplementary Materials, Section 1), and in view of parsimony, we tested a one-factor model with all the above mentioned tests as manifest indicators and academic achievement as latent factor. The final model showed a good fit (see online Supplementary Materials, Section 1), and the estimated academic achievement factor score was used in subsequent analysis.

##### Executive Functions

Executive functions were measured with a combination of performance-based tests and parent- and teacher rating scales.

*Performance-Based Executive Functions*. The performance-based measures entailed six subtests from the IDS-2 (Grob and Hagmann-Von Arx, 2018). The subtests Letter and Digit Recall and Picture Recognition were administered to measure verbal- and visuo-spatial working memory respectively. Fluency was measured during the subtest Naming Words. The subtest Divide Attention tapped into the ability to divide attention between different tasks, as well as into cognitive flexibility and working memory. Finally, the subtest Naming Animal Colors measured interference control, while the subtest Crossing Roads was used to assess problem solving- and planning skills, which are considered higher order EFs (Baggetta and Alexander, 2016). The test manual provides sufficient evidence for reliability, convergent-, and discriminant validity of these subtests (Grob and Hagmann-Von Arx, 2018).

Given the length of the total test battery, and the high demands EFs tasks pose on the motivation and concentration of a child (Hughes and Graham, 2002), we designed a planned missing design for the performance-based EFs tests (for the design specifications, see Supplementary Materials, Section 1; (Graham et al., 2006; Rhemtulla et al., 2016). Next, to validate that EFs could be represented by one latent factor, as suggested by the IDS-2 manual, we conducted a one-factor CFA with the standard scores of each subtest as manifest indicators. For model specifications and results per step of the model trimming process, see Supplementary Materials, Section 1. Based on model fit indices, a one-factor model, with the factor loadings of the subtests Picture Recognition and Naming Animals constrained to zero, was chosen as the final model. Factor scores were estimated from this model for use in subsequent analysis.

*Rating Scales of Executive Functions*. Furthermore, we used the Dutch version of the Behavior Rating Inventory of Executive Functioning (BRIEF) (Huizinga and Smidts, 2012) to assess the everyday behavioral manifestations of EF in the home (parent form) and educational (teacher form) environment of children. For the current study, all raw item scores were added to obtain a total score, which was converted to an age- and gender-corrected T-score. Good reliability was confirmed in a Dutch sample, which also provided sufficient evidence for convergent-, divergent-, and discriminant validity (Huizinga and Smidts, 2010).

##### Motor Skills

Both performance-based tests and a parental questionnaire were used to measure motor skills. First, we administered three psychomotor subtests from the IDS-2 (Grob and Hagmann-Von Arx, 2018), that is, Gross motor, Fine motor, and Visuomotor. The Fine motor and Visuomotor subtests are scored based on time to complete the task and quality of execution. Age-corrected *z*-scores were averaged to create a composite score for psychomotor skills. Good internal consistency has been shown, as well as convergent validity based on significant associations with performance on the MABC-2. Second, parents filled out the Dutch version of the Developmental Coordination Disorder Questionnaire (DCD-Q) (Schoemaker, 2003) which addresses the functional manifestations of motor coordination skills in daily live activities. Raw item scores were summed to create a total score, ranging from 15 to 75. Children with a score of equal to, or below 46 are classified as being at risk of motor coordination difficulties. Psychometric evaluation of the originally Canadian instrument, confirmed internal consistency and validity in a Dutch sample, and extended usefulness of this tool to an age range of 5- to 8-year-olds (Schoemaker et al., 2006). Given the significant correlation between the psychomotor and DCD-Q score (*r* = 0.38, *p* = 0.001), we created a composite motor score, by *z*-standardizing each score and subsequently taking the mean.

##### Socioemotional Behavior

In order to assess children’s socioemotional functioning, positive socioemotional skills and maladaptive behaviors were assessed based on performance-based tests and multi-informant ratings. Performance-based tests entailed three subtests from the IDS-2 (Grob and Hagmann-Von Arx, 2018), tapping into socioemotional competencies. The subtests (i.e., Emotion Recognition, Emotion Regulation, and Socially Competent Behavior) were combined into a Socioemotional Competencies score, by averaging the *z*-scores of the subtests. The composite score showed good internal consistency, as well as convergent validity with the SDQ peer problems scale.

Next to these performance-based tests, the Dutch versions of the Child Behavior Checklist (CBCL/6-18) and the Teacher Report Form (TRF) were administered to parents and teachers, respectively (Verhulst and van der Ende, 2013). These questionnaires are part of the Achenbach System of Empirically Based Assessment (ASEBA), and are designed to measure socioemotional problem behaviors that have been displayed by the child during the past 6 months. A Total Problems score was created by summing raw scores of all individual problem items. This total score was converted to age- and gender-corrected T-scores. The Dutch translations of the CBCL and TRF have been found to be valid and reliable (Ivanova et al., 2007a, b; Verhulst and van der Ende, 2013).

##### Classroom Behavior

The pre-requisite learning skills test [Leervoorwaarden Test (LVT); Scholte and van der Ploeg, 2011] taps into cognitive and socioemotional pre-requisite learning skills that can either support or hamper learning. These pre-requisite learning skills can be divided into direct and indirect pre-requisite learning skills. Direct pre-requisite learning skills pertain to an individual child’s classroom behavior, such as motivation and planning skills. Indirect pre-requisite learning skills affect learning achievements in an indirect manner, and consist of a child’s sociometric status, and relation to peers and the teacher. Three scale scores can be derived by summing up the raw scores of the corresponding items, that is, Direct Learning Conditions, Social Embeddedness, and Relations. Higher scores on these scales indicate less favorable pre-requisite learning skills. As performance on all scales has been found to be associated to gender, scale scores were *z*-standardized within subsamples of boys and girls. The LVT has been found to exhibit good psychometric properties in terms of reliability and validity (Scholte and van der Ploeg, 2011).

##### Creative Thinking

The Test of Creative Thinking-Drawing Production–form A (TCT-DP) (Urban and Jellen, 2010) was used to measure creative potential. Children were asked to complete an unfinished drawing, with no further instructions, except that they were only allowed to use a pencil. The unfinished drawing involved a standard form with some specific elements, such as a half square. A total raw score was obtained by summing scores that were given for 13 categories (e.g., “new elements” or “unconventionality”). Good interrater- and test–retest reliability has been confirmed (for an overview see Urban and Jellen, 2010; Willemsen et al., 2020). Given the novelty of this measurement, evidence on validity is still scarce. Still, one study found significant correlations between TCT-DP scores and tests of creative mathematics and -writing (Willemsen et al., 2020) in a Dutch 4th grade sample, which supports convergent validity.

### Procedure

This study was carried out in accordance with the recommendations of, and with approval from the Ethics Review Committee of the Department of Pedagogical and Educational Sciences, Faculty of Behavioural and Social Sciences, University of Groningen. All parents and teachers gave written informed consent in accordance with the Declaration of Helsinki. The data were collected by graduate students in Pedagogical and Educational Sciences, Psychology, and Human Movement Sciences. Before they were allowed to collect any data, they had to follow and pass an extensive training.

Data collection concerning school readiness indicators, consisted of two home sessions, each lasting 90–120 min, during which the children performed several motor, cognitive, and language tests as part of the MELLE-project. The assessments were videotaped for scoring purposes, and to allow for later review of the data and of fidelity in following testing procedures. Children were encouraged with stickers after every task. When necessary, breaks were used to maintain attention and motivation. After each assessment, children received a small gift and a diploma. Parents filled out questionnaires on their child’s development, behavior, and daily environment. In return, parents received a report with the test results of their child. To ensure confidentiality, data were entered and stored using a personalized study identifier.

School outcome data were collected during the spring term of a child’s first grade year, and followed a comparable procedure as during school readiness data collection. That is, measurements were administered during one home visit of 120 min, during which parents filled in the questionnaires described above. Parents received an information package for their children’s teacher and were asked to deliver this in person. Afterward, we approached teachers by mail, that contained a link to the questionnaires which were digitalized by means of Qualtrics. Before entering the actual questionnaires, teachers were asked to indicate having read all relevant information, and to consent to participation.

### Analytical Approach

Our data-analyses proceeded in two phases. First, we set out to identify school readiness profiles by performing a latent profile analysis (LPA) in Mplus Version 8.3 (Muthén and Muthén, 2017). Based on previous PC studies, we tested a range of one- to six-profile solutions. Details concerning model specification and sensitivity analysis are presented in the Supplementary Materials, Section 3. Readers unfamiliar with (the advantages and pitfalls of) mixture modeling are referred to the following sources, which cover these techniques from a more introductory and methodological perspective (Bauer, 2007; Samuelsen and Raczynski, 2013; Berlin et al., 2014; Lanza and Cooper, 2016; Nylund-Gibson and Choi, 2018). As recommended by Tein et al. (2013), we applied an iterative approach, by testing which indicators added significantly to profile separation. That is, we carried out Wald’s tests to examine mean differences between profiles for each school readiness indicator. Next, we removed those indicators that did not add significantly (i.e., omnibus test was not significant and/or was of a small effect size) to profile separation, reran all previous analysis steps, and compared results.

Model fit was evaluated by means of a combination of statistical model fit indices, classification accuracy, profile prevalence, and theoretical interpretability. As recommended, we aimed to choose the most parsimonious and conceptually sound model solution (Nylund et al., 2007). Regarding statistical fit indices, we inspected the Akaike’s Information Criterion (AIC), the Bayesian Information Criterion (BIC), and the Sample-Size Adjusted BIC (SS-ABIC) (Ram and Grimm, 2009). Lower values indicate a good balance between model fit and parsimony (Collins and Lanza, 2009). Additionally, we compared model solutions with *k* profiles to solutions with *k*-1 profiles by means of the Bootstrap Likelihood Ratio Test (BLRT), where a significant *p*-value supports selection of the model with *k* profiles. Classification accuracy was evaluated according to the entropy of the model (>0.80) and profile prevalence should be at least 5% for all profiles. After deciding on the best and final model, we saved each child’s most likely membership to be used in the subsequent analysis phase.

In the second analysis phase, we examined associations of the most likely profile membership to school outcomes (second research question). We conducted a *post hoc* power analyses, which revealed that the subsample that was used for these analyses was limited in terms of the power to detect a medium effect (0.30, α = 0.05) through inferential parametric ANOVAs. Instead, we carried out Monte Carlo permutation tests in R (R Studio, Version 1.03.1093). Permutation tests are specifically suited for small and unbalanced datasets such as ours (Kunnen, 2011). These tests explore the probability of randomly finding an effect size equal to or greater than the one actually found in the sample under study. This probability is expressed in a *p*-value (similar to inferential statistics), which is a close approximation of the exact *p*-value. That is, by repeatedly resampling data from the study population, and computing the relevant test statistic, a distribution of test statistics is generated that is based on the properties of the sample itself. Thus, irrespective of deviations from normality or the presence of outliers, this *p*-value will be reliable (Dugard et al., 2011). Additionally, by increasing the number of permutations, the chance of correctly rejecting the null hypothesis (in our case: a mean difference of zero) grows accordingly (Kunnen, 2011). Therefore, we used 9999 replications (i.e., permutations) in all permutation tests. To examine whether profiles differed in terms of first grade school outcomes, permutation one-way ANOVAs were carried out (wPerm, Version 1.0.1). *Post hoc* comparisons between profiles were conducted by means of pairwise permutation tests (rcompanion, Version 2.4.0), with a false discovery rate correction to avoid inflation of Type I error. *Post hoc* comparisons were carried out for all ANOVAs that resulted in a statistically significant omnibus test. Additionally, it has been found that non-significant omnibus tests can be accompanied with significant *post hoc* comparisons (Chen et al., 2018). Therefore, to avoid such instances of false-negatives, while at the same time only focusing on school outcomes with actual variance to explain, we also ran *post hoc* comparisons, for non-significant ANOVAs with at least a medium effect size.

## Results

### Preliminary Analyses

Missing data rates on school readiness indicators ranged from 5.5 to 58% (for an overview per variable, see Supplementary Table 2 .1). Most missing data occurred with the EFs performance-based tests and the Pseudowords Repetition task. Given a non-significant Little’s MCAR test (χ^2^ (702) = 721.27, *p* = 0.299), we considered the missing data mechanism of the school readiness data to be missing completely at random. School readiness missing data were handled by the application of Full Information Maximum Likelihood (FIML) estimation to the LPA in Mplus. Concerning school outcomes, data were missing between 2.1 and 44.7% (for an overview per variable, see Supplementary Table 2 .2). Most data were missing on all three teacher questionnaires. List wise deletion was applied by default during the permutation one-way ANOVAs.

Table 1 provides an overview of means, standard deviations and ranges for all school readiness indicator variables. Zero-order Pearson correlations between all school readiness indicators are presented in the online Supplementary Table 1.

**TABLE 1:** Means, standard deviations and range of school readiness indicators.

	***M***	***SD***	**Range**
***Executive functions***			
BRIEF-P inhibition^b^	49.22	8.99	34–72
BRIEF-P working memory^b^	50.70	9.76	36–83
Day/Night^a^	10.59	3.53	2–16
Hand tapping^a^	10.56	3.64	2–16
Head-Toes-Task^a^	16.14	3.79	6–20
Digit recall^a^	4.81	1.28	2–7
Corsi block^a^	3.72	1.45	0–6
***Language and emergent literacy skills***			
WPPSI-III-NL receptive vocabulary^c^	10.52	2.78	3–18
CB&WL picture naming (seconds)	122.62	39.11	52–236
STT pseudowords^a^	18.37	7.87	4–34
***Motor skills***			
MABC-2-NL manual dexterity^c^	10.81	3.09	5–19
MABC-2-NL aiming and catching^c^	10.73	3.03	5–19
MABC-2-NL balance^c^	9.49	2.96	5–18
***Socioemotional skills^a^***			
SDQ externalizing	4.43	2.93	0–12
SDQ internalizing	2.70	2.41	0–10
SDQ prosocial	7.83	1.51	3–10

*BRIEF-P = Behavior Rating Inventory of Executive Functioning-Pre-school; WPPSI-III-NL = Wechsler Pre-school and Primary Scale of Intelligence Third Edition Dutch version; CB&WL = Rapid Naming Test; STT = Schlichting Language Test; MABC-2-NL = Movement Assessment Battery for Children-2 Dutch version; SDQ = Strengths and Difficulties Questionnaire.*

*^a^Raw (scale) scores.*

*^b^T-scores (*M* = 50, *SD* = 10).*

*^c^Standardized score (*M* = 10, *SD* = 3).*

### Identifying School Readiness Profiles

#### Class Enumeration

**FIGURE 2:**
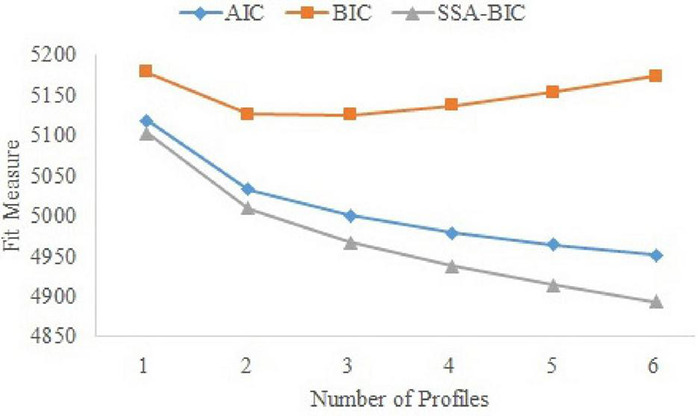
Elbow plot of latent profile analysis fit indices, that is, AIC = Akaike’s Information Criterion, BIC = Bayesian Information Criterion, and SSA-BIC = Sample-Size Adjusted BIC.

Replications of the best log likelihood and independence of school readiness indicators within profiles assured that no serious violations to the assumptions of (1) finding a global maximum and (2) conditional independence were present. Fit indices of the six profile solutions that we tested are presented in Table 2. Both the AIC and SSA-BIC decreased throughout the 6-profile solution; however the BIC was at its lowest for the 3-profile solution. Entropy was above 0.80 for all solutions, and in these instances the BIC should be privileged over the SSA-BIC (Diallo et al., 2017). Of note, the AIC and SSA-BIC tend to overestimate, whereas the BIC tends to underestimate the amount of profiles to be extracted (Nylund et al., 2007). The elbow plot (Figure 2) also showed a leveling off of the decrease of the AIC and SSA-BIC from a 4-profile solution onward. Thus, in combination, the statistical fit indices guided us toward a preliminary inspection of the 3- and 4-profile solutions. Both of these solutions were interpretable from a conceptual perspective, yet the BLRT revealed that the 4-profile solution fitted the data significantly better than the 3-profile solution. Moreover, the classification accuracy (as evaluated through the entropy) was slightly better for the 4-profile solution. Consequently, we decided upon the 4-profile solution as best representing the heterogeneity of school readiness in our sample.

**TABLE 2:** Absolute and relative model fit indices, entropy, and smallest profile size per profile solution.

***k***	***fp***	**AIC**	**BIC**	**SSA-BIC**	**BLRT (*p*)**	**Entropy**	**Prevalence smallest profile (%)**
1	24	5118.44	5178.44	5102.692	NA	NA	NA
2	37	5033.53	5126.02	5009.23	110.91 (<0.001*)	0.84	42 (47%)
3	50	5000.23	5125.27	4967.46	59.25 (<0.001*)	0.89	12 (13%)
4	63	4979.41	5136.89	4938.06	46.87 (<0.001*)	0.91	7 (8%)
5	76	4964.48	5154.46	4914.60	40.93 (<0.001*)	0.89	7 (8%)
6	89	4951.63	5174.11	4893.22	38.85 (0.10)	0.93	7 (8%)

*k = amount of profiles extracted; *fp* = number of free parameters; AIC = Akaike’s Information Criterion; BIC = Bayesian Information Criterion; SSA-BIC = Sample-Size Adjusted BIC; BLRT = Bootstrapped Likelihood Ratio Test.*

*significant *p*-value, indicating that this solution fits the data significantly better as compared to the *k*-1 solution.

#### Description of School Readiness Profiles

**FIGURE 3:**
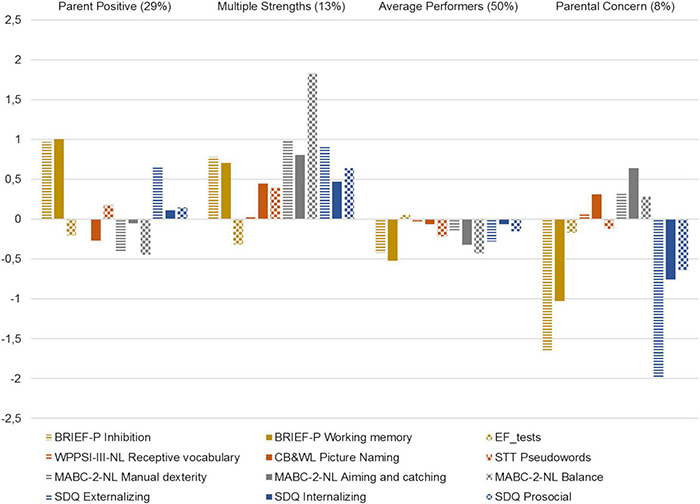
Patterns of mean z-standardized scores of children’s school readiness skills per profile. BRIEF-P = Behavior Rating Inventory of Executive Functioning-Pre-school; WPPSI-III-NL = Wechsler Pre-school and Primary Scale of Intelligence Third Edition Dutch version; CB&WL = Rapid Naming Test; STT = Schlichting Language Test; MABC-2-NL = Movement Assessment Battery for Children-2 Dutch version; SDQ = Strengths and Difficulties Questionnaire. All scores are put on the same metric, i.e., for all measures a higher score reflects better functioning and/or less problems.

Means of each school readiness indicator per profile are presented in Table 3. Figure 3 exemplifies each profiles’ specific pattern of relative strengths and weaknesses. The choice of names for each profile was based on features that stood out for that specific profile, and are not intended as a comprehensive description of school readiness performance within each profile. Furthermore, this study was aimed at school readiness at an age well before the entry of more formal schooling. As such, the focus was on school readiness skills that are emerging and could still follow diverse developmental paths. Therefore, we decided to avoid profiles names suggestive of a certain level of school readiness, as drawing any conclusions about the readiness of subgroups of children at this young age would feel premature. Children within the first profile (“Parent Positive”; 29%) were rated positively (i.e., above the sample average) by their parents concerning EFs and socioemotional behavior. This profile was further characterized by variability in scores on performance-based tests measuring motor and language/emergent literacy skills. For example, concerning emergent literacy, children in this profile scored above the sample mean on the non-word repetition task, whereas their results on the rapid naming task were below the sample mean. The second profile (“Multiple Strengths”; 13%) consisted of children showing strengths (i.e., performance above the sample mean) in multiple domains, especially with respect to motor skills. That is, unstandardized profile means on all MABC-2 subscales were above average if compared to this tests’ normative distribution for 3-year-olds. In addition, children within this profile scored above the sample mean in terms of emergent literacy, and were positively rated by their parents concerning EFs and socioemotional behaviors. These children did, however, score somewhat below the sample mean on performance-based EFs. Children within the third profile (“Average Performers”; 50%) did not show any distinct strengths or weaknesses, especially when compared to (the varied patterns of) the other profiles. Rather, these children displayed school readiness skill levels that were for the most part close to, or just below the mean of the total sample with regard to ball skills, balance, and parent-rated EFs and externalizing behaviors. Finally, children in the fourth profile (“Parental Concern”; 8%) stood out by a high level (>1.5 *SD* from the sample mean) of parental concern regarding EFs and externalizing problems. In fact, their EFs scores fell into the subclinical range and the SDQ externalizing scale scored at a clinical level. However, they displayed slightly above average performance on specific motor and language skills.

**TABLE 3:** School indicator means (standard error) per profile based on most likely class membership, and between-profile comparisons.

**Indicator**	**Parent Positive (26/29%)**	**Multiple strengths (12/13%)**	**Average Performers (45/50%)**	**Parental Concern (7/8%)**	**Mean differences**
BRIEF-P inhibition^a^	40.47 (1.48)	42.16 (1.83)	53.02 (1.13)	64.16 (1.41)	**1, 2 < 3, 4 & 3 < 4**
BRIEF-P working memory^a^	40.88 (0.85)	43.79 (2.01)	55.82 (1.72)	60.76 (1.12)	**1, 2 < 3, 4 & 3 < 4**
EF_tests^b^	−0.03 (0.21)	−0.06 (0.25)	0.04 (0.13)	−0.02 (0.21)	na
WPPSI-III-NL receptive vocabulary^b^	10.53 (1.14)	10.61 (0.66)	10.44 (0.61)	10.74 (1.22)	na
CB&WL picture naming^a^	133.24 (12.35)	105.07 (8.27)	125.20 (7.69)	110.47 (9.95)	1 > 2 & 2 < 3
STT Pseudowords^b^	19.77 (4.22)	21.46 (1.83)	16.65 (1.97)	17.42 (7.25)	2 > 3, 4
MABC-2-NL manual dexterity^b^	9.52 (0.68)	13.89 (0.80)	10.32 (0.52)	11.83 (1.02)	1 < 2, 4 & 2 **> 3,** 4 & 3 < 4
MABC-2-NL aiming and catching^b^	10.58 (0.64)	13.17 (0.93)	9.76 (0.37)	12.68 (1.48)	1 **< 2,** 4 & **2 > 3** & 3 < 4
MABC-2-NL balance^b^	8.17 (0.30)	14.90 (0.57)	8.23 (0.29)	10.32 (0.70)	**1 < 2, 4 & 2 > 3, 4 & 3 < 4**
SDQ externalizing^a^	2.50 (0.80)	1.75 (0.46)	5.25 (0.31)	10.26 (0.47)	**1 < 3, 4 & 2 < 3, 4 & 3 < 4**
SDQ internalizing^a^	2.43 (0.64)	1.56 (0.33)	2.86 (0.39)	4.52 (1.34)	1, 3 < 4 & 2 < **3**, 4
SDQ prosocial^b^	8.05 (0.41)	8.80 (0.44)	7.60 (0.26)	6.86 (0.34)	**2 > 3, 4**

*BRIEF-P = Behavior Rating Inventory of Executive Functioning-Pre-school T-scores; EF_tests = executive functions performance-based factorscore; WPPSI-III-NL = Wechsler Pre-school and Primary Scale of Intelligence Third Edition Dutch version, standardized score; CB&WL = Rapid Naming Test, raw score (seconds); STT = Schlichting Language Test, raw score; MABC-2-NL = Movement Assessment Battery for Children-2 Dutch version, standardized scores; SDQ = Strengths and Difficulties Questionnaire, raw scale scores. All mean differences are of a medium or large effect size (Cohen’s d); comparisons in bold are also significant at an alpha level of 0.05.*

*^*a*^Higher scores indicate more problems or a lower skill level.*

*^*b*^Higher scores indicate a higher skill level.*

#### Profile Separation

*Post hoc* Wald tests revealed that profiles were distinguishable on most school readiness indicators, except for the performance-based EFs and language scores (see Table 3). Line graphs of each profile, overlaid into one figure, are presented in the online Supplementary Materials, Section 3. The pattern of results from the *post hoc* comparisons showed that subgroups of children from the four profiles differed from each other in complex ways. As a sensitivity check, we reran all models without the (statistically) non-separating indicators, and found very similar results (detailed results available in online Supplementary Materials, Section 3). That is, fit indices were comparable, and even more supportive of a 4-profile solution. Model results were also very much alike, both in terms of profile prevalence, as well as in terms of school readiness indicator means per profile. Finally, the most likely profile membership was the same for each child in both the original and the adjusted (i.e., without the non-separating indicators) 4-profile solution. All the different steps to examine the profile structure supported the robustness of the four distinct school readiness profiles.

### Differences Across Profiles on School Outcome Variables

Differences between school readiness profiles with regard to first grade school outcome measures are presented in Table 4. Effect sizes per pairwise comparison are presented in the online Supplementary Table 4. *Post hoc* comparisons revealed mean differences with a medium or large effect size for half of our school outcome variables: academic achievement, parent- and teacher-rated EFs, motor skills, parent-rated socio-emotional functioning, and teacher-rated direct pre-requisite learning skills. School outcome scores showed mixed-level patterns per profile, i.e., indicating both relative strengths and weaknesses in children’s performance. Some trends can be discerned from the *post hoc* comparisons, which often resembled the performance patterns found among the school readiness indicators. Note that when discussing results, “range” refers to the range of scores of the total sample of the present study. “Higher” refers to higher scores or less problems for all school outcomes.

**TABLE 4:** Means, standard deviations, and one-way ANOVA statistics for school outcomes among school readiness profiles.

**Variable**	**Overall**		**Profile 1**		**Profile 2**		**Profile 3**		**Profile 4**		**ANOVA**	
	***M***	***SD***	***M***	***SD***	***M***	***SD***	***M***	***SD***	***M***	***SD***	***F* ratio**	**ω^2^**
**AA (*n* = 46)**	0.21	0.67	0.43_a_	0.75	0.57_a_	0.52	−0.04_b_	0.61	0.56_a_	0.33	2.70^†^	0.10
EFs												
EF (*n* = 45)	0.18	0.76	0.40	0.82	0.07	0.83	0.06	0.76	0.35	0.10	0.62	0.03
**BRIEF parent form (*n* = 44)**	39.11	8.39	35.33_a_	9.15	32.33_a_	5.57	42.35_b_	7.37	43.00_b_	3.61	4.26*	0.18
**BRIEF teacher form (*n* = 26)**	40.08	4.65	36.80_a_	2.17	38.33_ab_	3.22	41.50_b_	5.16	39.50_b_	2.12	1.58	0.06
**Motor (*n* = 38)**	0.09	0.84	−0.02_a_	0.65	0.80_b_	0.73	−0.11_a_	0.86	1.00_b_	0.09	2.75^†^	0.12
Socioemotional skills												
IDS-2 SEC (*n* = 42)	10.17	1.72	10.55	2.75	10.21	2.78	9.90	1.56	10.63	3.32	0.42	0.04
**CBCL (*n* = 47)**	48.13	9.30	46.38_a_	9.31	38.50_b_	8.07	50.28_ac_	8.08	57.00_c_	7.00	4.48**	0.18
TRF (*n* = 31)	46.19	7.33	45.14	8.07	42.25	12.28	47.50	6.26	46.00	1.41	0.60	0.04
Classroom behavior												
**LVT-Direct Learning Conditions (*n* = 28)**	–0.16	0.98	−0.14_ab_	1.21	−0.48_a_	1.02	−0.14_ab_	0.99	0.25_b_	0.08	0.24	0.09
LVT-Social Embeddedness (*n* = 28)	–0.18	1.01	0.01_a_	0.62	−0.05_a_	1.15	−0.35_a_	1.09	0.46_a_	1.42	0.50	0.06
LVT-Relations (*n* = 28)	–0.28	0.51	–0.42	0.10	0.05	0.94	–0.29	0.49	–0.39	0.22	0.73	0.03
TCT-DP (*n* = 39)	13.49	7.46	10.00	5.16	14.60	5.18	15.74	8.83	12.00	4.24	1.70	0.04

*AA = Academic achievement factor score; EFs = Executive functions; EF = Executive functions factor score; BRIEF = Behavior Rating Inventory of Executive Functioning, parent and teacher total T-score; IDS-2 SEC = Intelligence- and Developmental Scales Second Version Socioemotional Competencies standard score; CBCL = Child Behavior Checklist Total Problems T-score; TRF = Teacher Report Form Total Problems T-score; LVT = Leervoorwaarden test, all subscales concern gender-corrected *z*-standardized scores; TCT-DP = Test of Creative Thinking–Drawing Production total raw score; ANOVA = analysis of variance. Variables in bold represent school outcomes that differed between (some of) the profiles.*

Profile 1 = Parent Positive; Profile 2 = Multiple Strengths; Profile 3 = Average Performers; Profile 4 = Parental Concern. Profile means that do not share a subscript differed significantly at α = 0.05 and/or with at least a medium effect size (ω^2^ > 0.3) on the specific school outcome, according to permutation *post hoc* comparisons with a false discovery rate correction. ^†^*p* < 0.10; *p < 0.05; **p < 0.01.

First, children from the “Parent Positive” profile were also rated in the higher range (in other words, less problems) by their parents concerning first grade EFs and socioemotional behavior. Additionally, these children performed in the lower range with regard to motor skills, and in-between other profiles in terms of direct pre-requisite learning skills. Second, the “Multiple Strengths” group scored in the highest range for almost all first grade school outcomes. Only teacher-rated EFs fell in-between those of the other profiles. Notably, academic achievement of the children in this seemingly high functioning profile was similar to the children in both the “Parent Positive” and the “Parental Concern” profiles. Third, the school outcome score pattern of the “Average Performers” was somewhat flat, showing no distinct high or low performance, which is comparable to their school readiness profile. Overall, their scores were situated in the middle- (parent-rated socioemotional behavior, and direct pre-requisite learning skills), or in the lower range (academic achievement, parent- and teacher-rated EFs, and motor skills). Finally, parents of the children in the “Parental Concern” profile showed less concern about first grade (as compared to 3-year-old) EFs and socioemotional behavior, albeit that first grade parent ratings of these children were still in the lowest range in our sample. In contrast, these children performed in the higher range concerning academic achievement and motor skills.

## Discussion

### Main Findings and Interpretation

The aim of this study was to identify subgroups of children that show distinct patterns of school readiness skills, at an age where there is still time to enhance each child’s chances to thrive in school. Our PC approach revealed four profiles that could be distinguished based on parent rated EFs and socioemotional behavior, motor skills, and to a lesser extent by emergent literacy skills. The school readiness skills conjointly shaped first grade school outcomes, in profile-specific ways.

Our first aim was to identify school readiness profiles by incorporating EFs, language and emergent literacy skills, motor skill, and socioemotional behavior. We identified four profiles; a “Parent Positive,” a “Multiple Strengths,” an “Average Performers,” and a “Parental Concern” profile. The number of profiles that we found aligned with the number found in previous PC studies that also included motor skills (Hair et al., 2006; Halle et al., 2012; Quirk et al., 2013; Tavassolie et al., 2020). Concerning the specific pattern of each profile, it is difficult to make precise comparisons between studies, given the variety of measurements and information sources used. Nevertheless, some interesting trends are worth mentioning when contrasting study-specific profiles on a coarser level. Both Mascareño et al. (2014) and Martarelli et al. (2018) found a profile similar to our “Multiple Strengths” profile. That is, children in such a profile scored above average on (almost) all school readiness indicators in comparison to the rest of the sample. Potentially, these children represent a subgroup that is often referred to as “high ability” or “developmentally advantaged” (Kettler et al., 2017). Unfortunately, such children might not be able to reach their full potential, as this is a group of children that is often overlooked in early childhood education policies (Gallagher, 2007; Pfeiffer and Petscher, 2008). If developmentally advantaged children are not identified as such by (pre-school) teachers, underestimation in combination with a non-tailored curriculum might result in underperformance, or even disruptive behaviors (Hodge and Kemp, 2006). In support of this rather undesirable outcome hypothesis, our academic achievement findings during the 1st grade indicated that children in the “Multiple Strengths” group did not substantially outperform children in all the other profiles. Alternatively, the first grade performance profile of these children represents their actual potential, suggesting the existence of a group of developmentally advantaged children in areas other than the core academic domain.

Another commonality between our study and some previous school readiness studies, is the finding of a profile in which children are characterized by a distinct below-average socioemotional functioning (Hair et al., 2006; Halle et al., 2012). Particularly, children in our “Parental Concern” profile had clinical scores regarding externalizing behavior. Both Hair et al. (2006) and Halle et al. (2012) labeled these profiles as “risk profiles,” without being very explicit about why these children would be at risk, and at risk of what. Nonetheless, their concerns are not unwarranted as it has been shown that (externalizing) behavior problems early in life are often followed by less favorable school outcomes, or with even more detrimental outcomes such as school dropout during adolescence (Duncan and Magnuson, 2014). Alternatively, the “risk” label might be premature, if we consider how below average socioemotional functioning might affect school outcomes, *in combination with* skills in other school readiness domains, as will be discussed below.

Finally, in line with previous PC studies (Hair et al., 2006; Halle et al., 2012; Quirk et al., 2016; Tavassolie et al., 2020), we found profiles that were characterized by a mixed pattern of school readiness skills in terms of performing below, at or above the sample average. While this could be an actual reflection of each child’s relative strengths and weaknesses, an alternative explanation concerns the discontinuous nature of early childhood development, and warrants caution when relying on a cross-sectional design. Indeed, early childhood development has been found to be discontinuous, in other words, displaying a variable or irregular rate of change (Darrah et al., 2003). Discontinuity is often accompanied by a lack of ipsative stability, meaning that developmental domains follow distinct developmental trajectories (Thelen and Smith, 2007). To explain the low degree of ipsative stability, a biological compensation mechanism has been proposed in the literature, which entails that limited developmental resources have to be divided between developmental domains (Ben-Sasson and Gill, 2014). It could therefore be that children are only able to develop in one (or only a few) school readiness skill at once which is at the expense of other skills. This might explain finding a mixed profile showing below and above average school readiness skills. Importantly, the notion of discontinuous development calls for future studies to employ a longitudinal design concerning the assessment of school readiness.

Additionally, and also concerning our first research question, a major strength of our study design is the incorporation of motor skills as part of school readiness profiles. Our findings suggest that motor skills can be considered as a distinguishing feature between school readiness profiles. That is, we found between-profile mean differences for all three indicators of the motor domain, and most pronounced for balance skills (i.e., all balance mean differences had a large effect size). The nature of between profile differences was highly similar for all three motor indicators. For example, children within the “Multiple Strengths” profile could be distinguished from children within all other profiles on manual dexterity, aiming and catching, and balance skills. Most previous studies that included motor skills did so as part of an overall physical health indicator (Hair et al., 2006; Halle et al., 2012), or only compared profiles on a mean total score of all school readiness indicators. Based on their findings, no conclusions on the *specific* contribution of motor skills to school readiness could be inferred. To the best of our knowledge, we are one of the first to clearly underscore that motor skills should not be overlooked when assessing a child’s school readiness from a PC approach.

The importance of motor skills also becomes apparent when taking a closer look at how school readiness profiles were associated with school outcomes. For example, the “Parental Concern” profile could be considered as a somewhat troubling profile, given that many of the parent ratings were in a subclinical range. At the same time, this profile was characterized by relatively strong motor skills. Importantly, these children did not underperform compared to their peers in terms of first grade academic achievement, and were no longer rated at (sub)clinical level by their parent (nor by their teachers) on EFs or socioemotional skills. This specific pattern of findings might be exemplary of the long-term compensation mechanism, which we already described in the Introduction. Based on our findings, we argue that this mechanism might involve the idea that age-appropriate gross motor skills facilitate social participation (Emck et al., 2009). Schoolyard games and sport activities often involve gross motor skills such as running, jumping, aiming and catching. Children who are better equipped with respect to such skills, may be more likely to engage in such group activities (Cameron et al., 2016). As a result, they become part of a positive participation cycle in which they have more opportunities to practice and develop their socioemotional skills. Indeed, well-developed motor skills have been associated with positive first grade socioemotional adjustment (Bart et al., 2007).

With respect to the second research question, we found that school readiness profiles differed in mean scores on first grade academic achievement, parent- and teacher-rated EFs, motor skills, parent-rated socioemotional functioning, and pre-requisite learning skills. The pattern of mean differences was complex, suggesting that profiles could not be ranked from low too high in terms of school outcomes. Current findings also endorsed our implementation of a whole child perspective on measuring school outcomes. Had we solely focused on academic achievement, as in many previous studies, we might have concluded that our profiles largely lack predictive validity. That is, in the academic school outcomes only the “Average Performers” differed from children in the other three profiles. However, analyzing the pattern of mean differences with respect to the other school outcomes provides a more nuanced account of how school readiness skills conjointly affect school outcomes. Notably, there was no univocal ranking or ordering of profiles in terms of school outcomes. None of the profiles displayed lowest or highest scores on all, or at least the majority of, school outcomes. Amongst this complexity of findings, some trends deserve special mention.

First, we found that different starting positions in terms of school readiness can lead to similar outcomes. For example, the “Multiple Strengths” and “Parental Concern” profiles showed similar patterns of achievement in the academic and motor domains at first grade. This resonates with the concept of equifinality, which entails that a variety of initial conditions might result in the same end state through a myriad of pathways (Cicchetti and Rogosch, 1996). From this perspective, the developing child should be considered as an open system, which evolves in interaction with the environment. Moreover, children from different school readiness profiles are not predetermined to “end up” in different and fixed school outcome states.

A second noticeable finding concerns the performance of children within the “Average Performers” profile. While these children did not exhibit any distinct weaknesses (nor strengths) in terms of school readiness, they scored in the lower range on all school outcomes. One could argue that this is a relatively “unnoticed” group at home and in kindergarten. These could be the compliant, “average” scoring children, that subsequently do not receive any specific attention, for example through reinforcement or remediation efforts. While very tentative, this hypothesis finds some support in a study about teacher’s pedagogical practices (Thijs et al., 2006). Average children received less behavioral- and socioemotional support compared to their shy/inhibited or hyperactive peers. Another study (Waterhouse, 1995) found that average children (as identified by teachers) became largely invisible to their teachers, and subsequently ended up in teacher’s margins of attention. While “Average Performers” might fall short of a tailored pedagogical approach, it is concerning that they also did not have any relatively strongly developed compensatory skills.

### Limitations and Future Directions

We recognize several limitations that should be taken into consideration when interpreting our findings. First, our sample size might be considered as small for conducting LPA (Hickendorff et al., 2018). It is however very challenging to establish an adequate sample size, as this depends on many interacting factors, such as number and reliability of indicators (Lubke and Muthén, 2005). Inadequate sample sizes can lead to convergence issues, the inability to detect substantially important, yet low prevalent profiles, and unstable solutions (Nylund-Gibson and Choi, 2018). As for the former, we did not encounter any convergence issues, and concerning the latter we recommend replication studies to assess the robustness of our results. Furthermore, we tried to alleviate some of the potential issues related to small samples by using many, well-separating and reliable indicators, and by employing an iterative approach as recommended by Tein et al. (2013). Nonetheless, we cannot rule out the possibility that we lacked the power to identify a small, yet relevant school readiness profile. A second limitation concerns the generalizability of our results, which is limited to a low-risk and predominantly White sample. Third, our school outcome data-collection was limited to a single measurement occasion. Such a snapshot at a certain moment might reveal a crucial, yet temporary impression of school readiness. It is however, highly recommended to estimate the long-term predictive value of the school readiness profiles.

Finally, notwithstanding the merits of a PC approach, several drawbacks should be acknowledged concerning the corresponding analytical techniques. First, the process of class enumeration is a complex one, and very dependent on a scholar’s ability to combine detailed statistical information with sound substantive knowledge (Harring and Hodis, 2016). Arguably, this renders the decision process somewhat subjective. In addition, some methodologists have argued that applying mixture modeling will always lead to finding multiple latent profiles, even when none actually exist (Bauer, 2007). To alleviate this potential conundrum, we tried to formulate hypothesis concerning quantity and quality of profiles as specific as possible, and subjected different profile solutions to substantive interpretability. Moreover, the current mixture modeling options are mostly apt for theory development, while theory testing (e.g., confirming hypotheses concerning specific within-profile patterns) is challenging. Besides, our own results in combination with those of previous studies indicate that school readiness profile solutions are somewhat measurement dependent, which disputably questions the robustness and generalizability of PC findings.

In a previous section, we already argued in favor of future studies employing a longitudinal design for measuring school readiness profiles. A related issue concerns the stability of school readiness profiles. Questions left unanswered are if similar school readiness profiles emerge at different time-points, and how many and which children shift between profiles. Furthermore, we speculated on potential parenting mechanisms explaining some of the profile patterns and/or differential associations with school outcomes. This discussion highlights the importance of examining child development, and school readiness more specifically, from an ecological perspective (Bronfenbrenner and Morris, 2007). Future work should therefore examine associations of school readiness with the child’s proximal context, such as parental attitudes, their actual parenting practices, and parent-child interaction. Moreover, it should be explored whether (adverse) characteristics of the school and classroom moderate/mediate the association between a child’s school readiness and her/his subsequent school outcomes.

### Practical Implications

Several practical implications can be derived from the findings of the present study. First, it is important for all those involved in early childhood care and education to understand and assess a child’s functioning based on a combination of school readiness skills. Doing so, will not only provide a more accurate picture of a child’s development, but also help design a tailored approach to each child’s specific strengths and weaknesses profile. Furthermore, our results support the increasing attention to gross and fine motor skills in several national school readiness assessment policies (e.g., Rechtik, 2018; Koriakin et al., 2019). Including motor skills in any school readiness assessment is important not only because of their distinguishing potential, but also because relatively strong motor skills could be drawn upon as long-term compensation for less developed skills. Finally, we found some evidence of heterotypic stability of especially parent-rated EFs and -socioemotional behavior, and performance-based motor skills. Based on these findings, early childhood policies should particularly focus on and attend to early delays in these school readiness skills, as performance at 3-years-of-age is often reflected in similar performance in first grade. As measurement of these skills included multiple methods, and we cannot yet draw conclusions of the predictive validity of any of these methods, early childhood screening should also draw upon a mixed-sources approach.

## Conclusion

In a nutshell, the main finding of our study was the identification of four distinct school readiness profiles. Being one of the first to consider motor skills as part of school readiness, we showed that motor skills clearly distinguished between children in the four school readiness profiles. Moreover, motor skills might act as a protective factor for children with socioemotional and/or EFs problems. School readiness profiles were differentially related to school outcomes in intricate ways. We suggest that parents and teachers not only have an important role in identifying each child’s specific needs, but also in providing tailored guidance and support. Taken together, our findings highlight the importance of employing a multifaceted description of both a child’s school readiness, and her/his school outcomes. Insights stemming from the four school readiness profiles, and their associations with later school outcomes, could guide schools (and more generally speaking: early childhood education and care) in “being ready” for each child’s unique strengths and needs. Pedagogical approaches that are sensitive to each child’s specific needs, also of those children whose development is above average, are needed to provide fully inclusive learning environments.

## Data Availability Statement

The datasets presented in this article are not readily available because parental consent was only given for use of data by the researchers directly involved in the [MELLE] study. Requests to access the datasets should be directed to EK, e.kamphorst@rug.nl.

## Ethics Statement

The studies involving human participants were reviewed and approved by Ethics Review Committee of the Department of Pedagogical and Educational Sciences, Faculty of Behavioral and Social Sciences, University of Groningen. Written informed consent to participate in this study was provided by the participants’ legal guardian/next of kin.

## Author Contributions

EK was responsible for data collection, conducted the analysis, wrote the first version of manuscript, and edited the manuscript. GV was responsible for data collection and contributed to reviewing the manuscript. MC, AM, and SH supervised the study and contributed to reviewing and editing the manuscript. All authors contributed to the article and approved the submitted version.

## Conflict of Interest

The authors declare that the research was conducted in the absence of any commercial or financial relationships that could be construed as a potential conflict of interest.

## Publisher’s Note

All claims expressed in this article are solely those of the authors and do not necessarily represent those of their affiliated organizations, or those of the publisher, the editors and the reviewers. Any product that may be evaluated in this article, or claim that may be made by its manufacturer, is not guaranteed or endorsed by the publisher.
