# Supplementary material for: Emerging School Readiness Profiles: Motor Skills Matter for Cognitive- and Non-cognitive First Grade School Outcomes
Source: Front Psychol. 2021 Nov 23;12:759480. doi: 10.3389/fpsyg.2021.759480 (PMC8751626; doi:10.3389/fpsyg.2021.759480)
Supplement: Supplementary file 1 [file Data_Sheet_1.pdf]

Supplementary materials

Belonging to:

Kamphorst et al., 2021 Emerging School Readiness Profiles:

Motor Skills Matter for Cognitive and

Non-cognitive First Grade

School Outcomes. *Frontiers in Developmental Psychology*

S1: Specification of and results from data summarizing and planned missing design

As preparation for the decision process concering data summarizing we ran bivariate correlations between all school readiness indicators. These results are presented in Table S1 (see next page).

For all CFA's we found missingness with respect to manifest indicators to be either MCAR (for first grade performance-based EFs, as indicated by a non-significant Little's MCAR test [χ^2 (86) = 89.17, p = .39]) or MAR (for school readiness performance-based EF's and academic achievement. MAR was assumed in these cases as several observed variables (e.g., age) were found to be related to missingness on both EFs and academic achievement. Accordingly, missingsness was handled by means of Full Information Maximum Likelihood (FIML) in all CFA's.

For all CFA's, model fit was assessed as follows. Good model fit was indicated by a

Table S1.1 Model fit Indices Regarding Confirmatory Factor Analysis of School Readiness Executive Functions (EFs) Performance-based Tests

χ^2 (p)	5.33 (.38)
χ^2 /df	1.07
RMSEA	.03
TFI/CFI	.97/.94
SRMR	.08

Note. A one-factor model was tested with all five EFs performance-based tests loaded onto one latent EFs factor. No multivariate outliers were present according to non-significant Mahalanobis distances. RMSEA = root-mean-square error of approximation; TFI = Tucker Lewis index; CFI = comparative fit index; SRMR = standardized root-mean-square residual.

combination of a non-significant χ^2, a ratio of χ^2 to degrees of freedom (χ^2/df) ≤ 2, a Root Mean Square Error of Approximation (RMSEA) and Standardized Root Mean Square Residual (SRMR) < .08, and the Tucker-Lewis Fit Index (TFI) and Comparative Fit index (CFI) > .90. We used the robust maximum likelihood estimator (MLR) to account for deviations from normality by several of the EFs and academic achievement subtests. The latent factor metric was defined by fixing the factor variance to one in all models.

S1.1 CFA school readiness EFs performance-based tests

Table S1 Correlations with confidence intervals for school readiness indicators

Variable	1	2	3	4	5	6	7	8	9	10	11
1. BRIEF-P Inhibition											
2. BRIEF-P Working memory	.74**<br>[.62, .82]										
3. EF_tests	.02<br>[-.20, .25]	.04<br>[-.19, .26]									
4. SDQ Externalizing	.71**<br>[.58, .80]	.52**<br>[.35, .67]	-.06<br>[-.28, .17]								
5. SDQ Internalizing	.12<br>[-.10, .33]	.13<br>[-.09, .34]	.26*<br>[.03, .46]	.22*<br>[.00, .42]							
6.SDQ Prosocial	-.31**<br>[-.50, -.10]	-.21<br>[-.41, .01]	.02<br>[-.21, .25]	-.43**<br>[-.59, -.23]	-.03<br>[-.24, .19]						
7. MABC-2-NL Manual dexterity	.00<br>[-.22, .23]	.03<br>[-.19, .25]	.25*<br>[.02, .46]	-.13<br>[-.35, .10]	-.10<br>[-.33, .13]	.17<br>[-.06, .39]					
8. MABC-2-NL Aiming and catching	-.13<br>[-.34, .09]	-.22<br>[-.42, -.00]	-.04<br>[-.27, .19]	-.07<br>[-.29, .16]	.10<br>[-.13, .32]	.07<br>[-.16, .29]	.20<br>[-.02, .40]				
9. MABC-2-NL Balance	-.20<br>[-.42, .04]	-.19<br>[-.40, .05]	.05<br>[-.20, .29]	-.22<br>[-.44, .02]	-.21<br>[-.43, .03]	.18<br>[-.07, .40]	.57**<br>[.39, .71]	.31**<br>[.09, .51]			
10. WPPSI-III-NL Receptive vocabulary	-.03<br>[-.24, .19]	-.03<br>[-.24, .19]	-.06<br>[-.28, .17]	-.10<br>[-.32, .13]	-.10<br>[-.31, .13]	.25*<br>[.02, .44]	.10<br>[-.12, .32]	.23*<br>[.01, .42]	.02<br>[-.22, .25]		
11. STT Pseudowords	-.34*<br>[-.60, -.02]	-.25<br>[-.53, .08]	.45**<br>[.14, .68]	-.27<br>[-.55, .07]	-.07<br>[-.40, .27]	.23<br>[-.11, .52]	.54**<br>[.27, .74]	.12<br>[-.21, .43]	.33<br>[-.02, .61]	.14<br>[-.20, .45]	
12. CB&WL Picture Naming	-.03<br>[-.28, .21]	-.03<br>[-.28, .21]	-.14<br>[-.38, .11]	.03<br>[-.23, .28]	-.17<br>[-.40, .09]	.09<br>[-.17, .33]	-.18<br>[-.41, .07]	-.11<br>[-.35, .15]	-.20<br>[-.44, .07]	.06<br>[-.19, .31]	-.23<br>[-.53, .11]

Note. Values in square brackets indicate the 95% confidence interval for each correlation. The confidence interval is a plausible range of population correlations that could have caused the sample correlation (Cumming, 2014). BRIEF-P = Behaviour Rating Inventory of Executive Functioning-Preschool; EF_tests = executive functions performance-based factor score;WPPSI-III-NL = Wechsler Preschool and Primary Scale of Intelligence Third Edition Dutch version; CB&WL = Rapid Naming Test; STT = Schlichting Language Test; MABC-2-NL = Movement Assessment Battery for Children-2 Dutch version; SDQ = Strengths and Difficulties Questionnaire. * $p < .05$. ** $p < .01$.

S1.2 CFA first grade academic achievement

Table S1.2.1 Zero-order Correlations for Academic Achievement Variables

Variable	1	2	3	4	5	6	7
1. NLT-PAE	−						
2. IDS-LWD	−.37**	−					
3. IDS-TE	−.32**	.37**	−				
4. IDS-TR	-.22*	.44**	.25*	−			
5. Cito-DMT	.33*	−.24	−.26	−.09	−		
6. Cito-Spelling	.26	−.26	-.12	-.33*	.36*	−	
7. Cito-RW	.60**	−.59**	−.26	−54**	.25	.53**	−

Note. NLT-PAE = number line task- percentage absolute error; IDS = Intelligence and Developmental Scales; LWD = logical-mathematical thinking subtest; TE = expressive language subtest; TR = receptive language subtest; Cito = nationwide tests for monitoring yearly progress of Dutch primary school students; DMT = three minute reading test; RW = mathematical test.
*p < .05 ** p < .01.

The one-factor model with all academic achievement tests loaded onto one academic achievement factor showed a good overall model fit. Inspection of Mahalanobis distances identified 3 potential multivariate outliers, of which Cooks' distances were slightly over one, suggesting some influence of these outliers. Inspection of raw data revealed that these cases were valid (i.e., no error outliers). As a sensitivity check we reran the model with these potential outliers removed, and found no substantial differences concerning model fit and standardized factor loadings (see table S1.2.2). Subsequently, we used the model with outliers for subsequent analyses.

Table S1.2.2 Results Regarding One-factor Model CFA of School Outcomes Academic Achievement (N = 93)

	Model with outliers[a]	Model without outliers[b]
Fit Indices		
χ^2 (*p*)	18.83 (.17)	16.65 (.28)
χ^2 /df	1.35	1.19
RMSEA	.06	.05
TFI/CFI	.94/.92	.96/.95
SRMR	.076	.076
Standardized loadings		
NLT-PAE	-.53***	.58***
IDS-LWD	.66***	-.71***
IDS-TE	.54***	-.46***
IDS-TR	.56***	-.43**
Cito-DMT	-.33*	.33
Cito-Spelling	-.65***	.61***
Cito-RW	-.84***	.83***

Note. RMSEA = root-mean-square-error of approximation; TFI = Tucker Lewis Index; CFI = Comparative Fit Index; SRMR = Standardized Root Mean Square Residual; NLT-PAE = number line task- percentage absolute error; IDS = Intelligence and Developmental Scales; LWD = logical-mathematical thinking subtest; TE = expressive language subtest; TR = receptive language subtest; Cito = nationwide tests for monitoring yearly progress of Dutch primary school students; DMT = three minute reading test; RW = mathematical test.
[a] In this model all seven items of academic achievement were loaded onto one factor. [b] The same model was tested, but with outliers removed.
* p < .05 ** p < .01 *** p < .001.

S1.3 Planned missing design and CFA first grade performance-based EFs tests

As a result, such designs increase validity of measurement, because participants are prevented from being overburdened. We created three EF battery versions, such that the covariance coverage matrix did not include any zeros on the off-diagonals ((Silvia et al., 2014) see Table S1.3.1). These batteries were randomly distributed across children. The randomization of missingness enables reliable and unbiased FIML estimation methods. That is, to confirm that EF could be represented by one latent factor, as suggested by the IDS-2 manual, we conducted a one-factor CFA with the standard scores of each subtest as manifest indicators. As shown in table S4, the one factor model showed a good model fit, yet a TFI above one might suggest overfitting of the model (Kline, 2015). Therefore we trimmed the model, by constraining non-significant factor loadings to zero. Most fit indices indicated that this adjusted model fitted our data well, except from the SRMR. Based on the modification indices, we allowed the factor loading of ‘Crossing roads’ to be freely estimated. This is in line with the finding that only (and not the other 5) this subtest is aimed at tapping into higher-order EFs and might therefore add complementary to the overall EF latent factor (Baggetta and Alexander, 2016). This model resulted in an overall good model fit (see table S1.3.2).

Table S1.3.1. Covariance coverage matrix of performance-based first grade EFs tests in the proposed planned missing design.

	Set A (Letter and Digit Recall & Crossing Roads)	Set B (Naming Words & Divide Attention)	Set C (Picture Recognition & Naming Animal Colors)
Set A (Letter and Digit Recall & Crossing Roads)	.67		
Set B (Naming Words & Divide Attention)	.33	.67	
Set C (Picture Recognition & Naming Animal Colors)	.33	.33	.67

Table S1.3.2. Results of Confirmatory Factor Analysis of First Grade Executive Functions Performance-based Tests (N = 91)

Fit Indices	Model 1[a]	Model 2[b]	Model 3[c]
χ^2 (*p*)	4.14 (.90)	7.56 (.82)	6.20 (.86)
χ^2 /df	0.46	0.63	0.56
RMSEA	.00	.00	.00
TFI/CFI	1.45/1.00	1.31/1.00	1.36/1.00
SRMR	.06	.10	.08
Standardized loadings			
Letter and Digit recall	.56***	.47**	.53*
Picture Recognition	.11	na	na
Naming Words	.64***	.56***	.60***
Divide Attention	.80***	.94***	.86***
Naming Animal Colors	.28	na	na
Crossing Roads	.26	na	.21

Note. RMSEA = root-mean-square-error of approximation; TFI = Tucker Lewis Index; CFI = Comparative Fit Index; SRMR = Standardized Root Mean Square Residual.
[a] In model 1 all 6 performance-based executive functions (EFs) tests were loaded onto one EFs_test laten factor. [b] In model 2, the non-significant factor loadings from model 1 (‘Picture Recognition’, ‘Naming Animal Colors’, ‘Crossing Roads’) were constraint to zero. [c] In model 3, the factor loading of the subtest ‘Crossing roads’ was freely estimated again.
* p <.05 ** p < .01 *** p < .001

S2: Overview of missing data on school readiness and school outcome data

Table S2.1. Amount of Missing Data of School Readiness Indicators

Variable	Percentage missing data
Executive Functions	
BRIEF-P Inhibition	5.5
BRIEF-P Working Memory	5.5
Day/Night	44.0
Hand Tapping	45.1
Head-Toes-Task	37.4
Digit Recall	26.4
Corsi Block	37.4
Language and Emergent Literacy Skills	
WPPSI-III-NL Receptive Vocabulary	6.6
CB&WL Picture Naming (seconds)	27.5
STT Pseudowords	58.2
Motor Skills	
MABC-2-NL Manual Dexterity	11.0
MABC-2-NL Aiming and Catching	8.8
MABC-2-NL Balance	19.8
Socioemotional Skills	
SDQ Externalizing	8.8
SDQ Internalizing	8.8
SDQ Prosocial	8.8

Note. BRIEF-P = Behavior Rating Inventory of Executive Functioning-Preschool; WPPSI-III-NL = Wechsler Preschool and Primary Scale of Intelligence Third Edition Dutch version; CB&WL = Rapid Naming Test; STT = Schlichting Language Test; MABC-2-NL = Movement Assessment Battery for Children-2 Dutch version; SDQ = Strengths and Difficulties Questionnaire.

Table S2.2. Amount of Missing Data of First Grade School Outcomes

Variable	Percentage missing data
AA	2.1
EFs	
EF	4.3
BRIEF parent form	16.1
BRIEF teacher form	44.1
Motor	
Socioemotional skills	
IDS-2 SEC	9.7
CBCL	5.4
TRF	32.3
Classroom behavior	
LVT-Direct Learning Conditions	36.6
LVT-Social Embeddedness	38.7
LVT-Relations	36.6
TCT-DP	15.1

Note. AA = Academic achievement factor score; EFs = Executive functions; EF = Executive functions factor score; BRIEF = Behavior Rating Inventory of Executive Functioning, parent and teacher total T-score; IDS-2 SEC = Intelligence- and Developmental Scales Second Version Socioemotional Competencies standard score; CBCL = Child Behavior Checklist Total Problems T-score; TRF = Teacher Report Form Total Problems T-score; LVT = Leervoorwaarden test, all subscales concern gender-corrected *z*-standardized scores; TCT-DP = Test of Creative Thinking – Drawing Production total raw score.

S3: Specification of latent profile analysis

During all latent profile analyses, means were freely estimated within every profile, while variances were constrained to be equal. To ensure a stable solution, that is: avoid a local maximum, we carried out two runs for each model. For the first, we requested 100 sets of initial starting values, and 20 for the optimization stage of the maximum likelihood estimation. During the second run, we doubled these starting values, and checked the replication of the best log likelihood afterwards. Additionally, we verified the assumption of local independence, by inspecting independence of school readiness indicators within profiles. The seeds of each stable solution were specified in subsequent model runs, using the 'OPTSEED' option of M*plus*. In these subsequent runs we requested for the parametric bootstrapped likelihood ratio test. Again, the number of starting values were doubled to check if results were sensitive to the number of random starts.

Figure S3. Patterns of mean z-standardized scores of children's school readiness skills per profile.

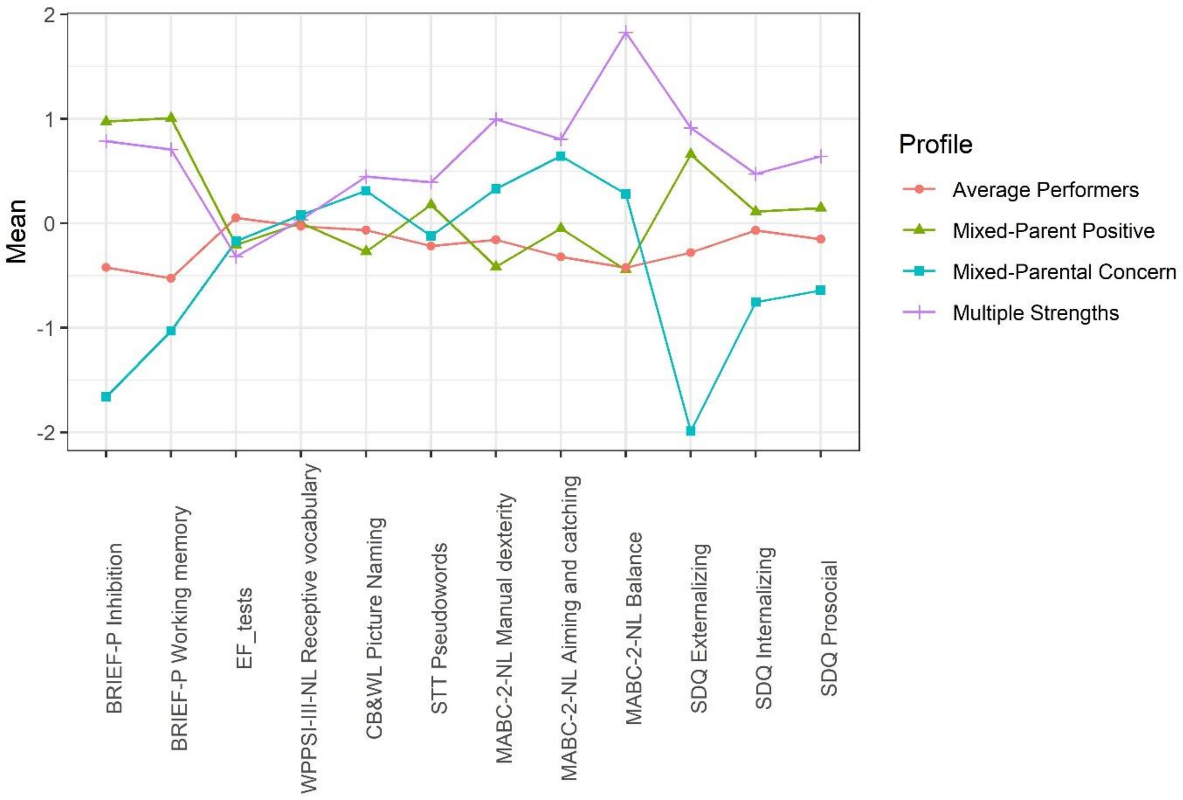

Results of sensitivity analyses

As recommended by Tein et al. (2013), we applied an iterative approach, by testing which indicators added significantly to profile separation. That is, we carried out Wald's tests to examine mean differences between profiles for each school readiness indicator. Next, we removed those indicators that did not add significantly (i.e., omnibus test was not significant and/or was of a small effect size) to profile separation, and reran all previous analysis steps. The model fit indices, and the profile means for the 3- and 4-profile solutions (named 'alternative model') are presented in tables S3.1, 3.2 and 3.3 respectively.

Table S3.1 Absolute and Relative Model fit Indices, Entropy, and Smallest Profile Size per LPA Solution of Alternative Model

k	*fp*	AIC	BIC	SSA-BIC	BLRT (*p*)	Entropy	Prevalence smallest profile (%)
1	20	4521.14	4571.14	4508.02	na	na	na
2	31	4432.38	4509.88	4412.04	110.76 (<.001*)	.83	47
3	42	4396.44	4501.44	4368.88	57.94 (<.001*)	.88	12
4	53	4370.36	4502.85	4335.58	48.08 (<.001*)	.91	7.8
5	64	4357.59	4517.58	4315.59	34.77 (<.001*)	.87	7.8
6	75	4348.07	4535.56	4298.85	31.52 (.19)	.90	7.8

Note. *k*=amount of profiles extracted; fp-number of free parameters; AIC = Akaike's Information Criterion; BIC = Bayesian Information Criterion; SSA-BIC = Sample-Size Adjusted BIC.
*significant *p*-value, indicating that this solution fits the data significantly better as compared to the *k*-1 solution.

Table S3.2 Indicator Means per Profile Based on most Likely Class Membership for Three-profile Solution of Alternative Model

Indicator	Profile 1 (31/ 34.4%)	Profile 2 (47/ 52.2%)	Profile 3 (12/ 13.3%)
MABC-2-NL Manual dexterity	9.49	10.66	13.87
MABC-2-NL Aiming and catching	10.27	10.31	13.16
MABC-2-NL Balance	8.21	8.56	14.88
BRIEF-P Inhibition	42.55	55.36	42.18
BRIEF-P Working memory	41.69	58.29	43.79
STT Pseudowords	19.57	16.41	21.53
CB&WL Picture Naming	133.32	121.34	105.18
SDQ Externalizing	3.28	6.03	1.76
SDQ Internalizing	2.61	3.09	1.57
SDQ Prosocial	7.99	7.43	8.81

BRIEF-P = Behaviour Rating Inventory of Executive Functioning-Preschool T-scores; EF_tests = executive functions performance-based factorscore ; CB&WL = Rapid Naming Test, raw score (seconds); STT = Schlichting Language Test, raw score; MABC-2-NL = Movement Assessment Battery for Children-2 Dutch version, standardized scores; SDQ = Strengths and Difficulties Questionnaire, raw scale scores.

Table S3.3 Indicator Means per Profile Based on most Likely Class Membership for Four-profile Solution of Alternative Model

Indicator	Profile 1 (26/ 28.9%)	Profile 2 (45/ 50%)	Profile 3 (7/ 7.8%)	Profile 4 (12/ 13.3%)
MABC-2-NL Manual dexterity	9.53	10.32	11.84	13.91
MABC-2-NL Aiming and catching	10.58	9.76	12.67	13.17
MABC-2-NL Balance	8.17	8.23	10.32	14.89
BRIEF-P Inhibition	40.47	53.03	64.17	42.18
BRIEF-P Working memory	40.91	55.84	60.77	43.81
STT Pseudowords	19.74	16.66	17.43	21.46
CB&WL Picture Naming	132.89	125.32	110.44	105.12
SDQ Externalizing	2.50	5.25	10.25	1.75
SDQ Internalizing	2.44	2.85	4.51	1.55
SDQ Prosocial	8.05	7.60	6.86	8.81

BRIEF-P = Behaviour Rating Inventory of Executive Functioning-Preschool T-scores; EF_tests = executivefunctions performance-based factorscore ; CB&WL = Rapid Naming Test, raw score (seconds); STT = Schlichting Language Test, raw score; MABC-2-NL = Movement Assessment Battery for Children-2 Dutch version, standardized scores; SDQ = Strengths and Difficulties Questionnaire, raw scale scores.

Section 4: Effect sizes for post-hoc pairwise comparisons of profile means on school outcomes

Table S4 Effect sizes (ω^2) for post-hoc pairwise comparisons of school outcomes mean differences

Variables	1-2	1-3	1-4	2-3	2-4	3-4
AA (n = 46)	.10	.31	.08	.37	.01	.30
EFs						
BRIEF parent form (n=44)	.18	.36	.34	.46	.62	.03
BRIEF teacher form (n = 26)	.20	.29	.34	.19	.18	.11
Motor (n = 38)	.42	.05	.44	.36	.15	.32
Socioemotional skills						
CBCL (n = 47)	.38	.22	.42	.47	.64	.26
Classroom behavior						
LVT-Direct Learning Conditions (n = 28)	.11	.00	.12	.11	.34	.11
LVT-Social Embeddedness (n = 28)	.03	.13	.19	.09	.20	.19

Note. AA = Academic achievement factor score; EFs = Executive functions; BRIEF = Behavior Rating Inventory of Executive Functioning, parent and teacher total T-score; IDS-2 SEC = Intelligence- and Developmental Scales Second Version Socioemotional Competencies standard score; CBCL = Child Behavior Checklist Total Problems T-score; LVT = Leervoorwaarden test, all subscales concern gender-corrected *z*-standardized scores; 1-2= comparison of profile 1 and 2 etc,; 1 = Parent Positive; 2 = Multiple Strengths; 3 = Average Performers; 4 = Parental Concern.

References

Baggetta, P., and Alexander, P. A. (2016). Conceptualization and Operationalization of Executive Function. *Mind Brain Educ.* 10, 10–33. doi:https://doi.org/10.1111/mbe.12100.

Kline, R. B. (2015). "Analysis of Confirmatory Factor Analysis Models," in *Principles and Practice of Structural Equation Modeling, Fourth Edition* (New York, UNITED STATES: Guilford Publications), 300–337. Available at: http://ebookcentral.proquest.com/lib/rug/detail.action?docID=4000663 [Accessed July 8, 2021].

Silvia, P. J., Kwapil, T. R., Walsh, M. A., and Myin-Germeys, I. (2014). Planned missing-data designs in experience-sampling research: Monte Carlo simulations of efficient designs for assessing within-person constructs. *Behav. Res. Methods* 46, 41–54. doi:10.3758/s13428-013-0353-y.

Tein, J.-Y., Coxe, S., and Cham, H. (2013). Statistical Power to Detect the Correct Number of Classes in Latent Profile Analysis. *Struct. Equ. Model. Multidiscip. J.* 20, 640–657. doi:10.1080/10705511.2013.824781.
